# Planned and Unplanned Sarcoma Resections: Comparative Analysis of Local Recurrence, Metastasis, and Mortality

**DOI:** 10.3390/cancers16193408

**Published:** 2024-10-07

**Authors:** Kim N. Nydegger, Timothy T. A. F. Obergfell, Philip Heesen, Georg Schelling, Gabriela Studer, Beata Bode-Lesniewska, Bruno Fuchs

**Affiliations:** 1 Faculty of Health Sciences and Medicine, University of Lucerne, Frohburgstrasse 3, 6002 Luzern, Switzerland; 2 Sarkomzentrum, Kliink für Orthopädie und Unfallchirurgie, LUKS University Hospital, Luzerner Kantonsspital, 6000 Lucerne, Switzerland; 3 Medical Faculty, University of Zurich, 8032 Zurich, Switzerland; 4 Sarkomzentrum KSW, Klinik für Orthopädie und Traumatologie, Kantonsspital Winterthur, 8400 Winterthur, Switzerland

**Keywords:** sarcoma, planned excision/resection, unplanned excision/whoops/resection, surgical outcomes, metastasis, cancer-specific mortality, tumor characteristics, early detection, predictive tools, sarcoma management

## Abstract

**Simple Summary:**

Sarcomas are rare and diverse tumors that develop in various tissues like bone, muscle, and fat. This study examines the outcomes of two types of surgical removal of these tumors: planned resections (PEs), where the tumor is diagnosed a-priori and surgically removed with a plan, and unplanned resections (UEs), where the tumor is removed without prior diagnosis. We found that planned surgeries, while better at completely removing the tumor initially, are associated with more advanced tumors that have a higher chance of spreading and leading to cancer-related deaths. On the other hand, unplanned surgeries tend to have higher chances of the tumor coming back locally but do not significantly affect long-term survival. These findings highlight the importance of early detection and timely treatment at specialized centers to improve patient outcomes. Our research aims to help develop better tools for predicting and managing sarcomas to enhance patient care.

**Abstract:**

Background: Sarcomas, a diverse group of malignant tumors arising from mesenchymal tissues, pose significant diagnostic and therapeutic challenges. This study compares the outcomes of planned resections (PEs) and unplanned resections (UEs) to inform better clinical practices. Methods: Data were analyzed from the Swiss Sarcoma Network (SSN), including patients with soft tissue and bone sarcomas treated at two major hospitals. This study utilized logistic regression and Cox regression models to examine the odds of UEs and their impact on local recurrence-free survival. Results: Among 429 patients registered by SSN members, 323 (75%) underwent PEs and 106 (25%) experienced UEs. PEs were associated with significantly larger tumors (94 mm vs. 47 mm, *p* < 0.001) and higher-grade tumors (Grade 3: 50.5% vs. 37.4%, *p* = 0.03). Despite achieving superior resection margins (R0: 78.8% vs. 12.6%, *p* < 0.001), PEs showed higher metastasis rates at follow-up (31.0% vs. 10.4%, *p* < 0.001) and greater cancer-specific mortality (16.7% vs. 6.6%, *p* = 0.01). UEs, while linked to higher local recurrence, did not significantly affect metastasis-free survival (MFS) or overall survival (OS). Conclusions: PEs achieve superior immediate surgical outcomes but are linked to higher metastasis and cancer-specific mortality due to the advanced stage of tumors. UEs, while associated with higher local recurrence rates, do not significantly impact MFS or OS. Early detection, comprehensive diagnostics, and timely referrals to specialized sarcoma hubs are essential to avoid UEs and reduce metastatic risk. Future research should focus on developing diagnostic tools using individual tumor and patient characteristics to improve sarcoma management.

## 1. Introduction

Sarcomas are a diverse group of malignant tumors arising from mesenchymal tissues, including bone, muscle, fat, and connective tissues [1]. Due to their rarity and heterogeneous nature, sarcomas present significant diagnostic and therapeutic challenges [2]. Surgical resection remains the cornerstone of sarcoma management, with the success of surgical outcomes heavily influenced by tumor characteristics such as size, grade, and anatomical location [3].

Unplanned resections (UEs) often occur when a sarcoma is not initially suspected, leading to suboptimal surgical outcomes [4,5,6,7]. These unplanned surgeries typically involve incomplete resections and higher rates of local recurrence [8]. Conversely, planned resections (PEs) are performed with a prior diagnosis of sarcoma, allowing for meticulous surgical planning and often achieving better immediate resection margins (R0 status). Our previous study highlighted that while unplanned resections are associated with higher local recurrence (LR) rates, there were no significant differences in metastasis-free survival (MFS) and overall survival (OS) between UE and PE groups [9]. This underscores the complexity of sarcoma management and the critical need for proper diagnosis and referral.

The early detection of sarcomas is crucial for optimal treatment planning. Proper diagnostic imaging and early referral to specialized sarcoma centers can significantly improve patient outcomes [10,11,12,13]. Early detection facilitates PEs, which are critical for achieving complete tumor removal and minimizing recurrence.

Several factors influence the probability of a sarcoma resection being planned or unplanned. Tumor size, grade, and anatomical location, as well as patient characteristics such as age and gender, play pivotal roles [14]. While composite predictive tools like Sarculator, which estimates survival rates, can aid clinicians in making informed decisions after a sarcoma is diagnosed, focusing on individual parameters provides more straightforward and actionable insights for early detection and intervention [15].

Despite the advantages of PEs in achieving complete tumor removal, the more advanced stage and aggressive nature of tumors requiring PEs may result in higher rates of metastasis and cancer-specific mortality [16,17,18]. This study aims to expand upon previous findings by further elucidating the differences in outcomes between PE and UE sarcoma resections, particularly addressing the ongoing debate in the literature about the impact of UEs on MFS and OS [19]. Understanding these differences is essential for improving early detection and optimizing referral practices, ultimately enhancing patient care and outcomes.

The objective of this study is to elucidate the differences in outcomes between PE and UE sarcoma resections and identify key predictive factors based on individual tumor and patient characteristics. By enhancing our understanding of these factors, this research aims to inform clinical practice and improve the management of sarcoma patients, ultimately leading to better survival rates and quality of life.

## 2. Materials and Methods

### 2.1. Data Source and Collection

Data from the Swiss Sarcoma Network (SSN) (https://www.swiss-sarcoma.net/ (accessed on 6 October 2024)) data warehouse were analyzed [20,21]. This network longitudinally collects data from patients with soft tissue and bone tumors, establishing a multicenter, prospective real-world/time data repository of high quality [22,23]. The current dataset is housed on Adjumed.ch (Adjumed Services AG, Zurich, Switzerland; www.adjumed.ch, accessed on 10 October 2023) and is expected to transition to the Sarconnector^®^ (BF&PH, Zurich, Switzerland), a dynamic real-world/time data warehouse for automated analysis [20].

### 2.2. Study Population

**Figure 1:**
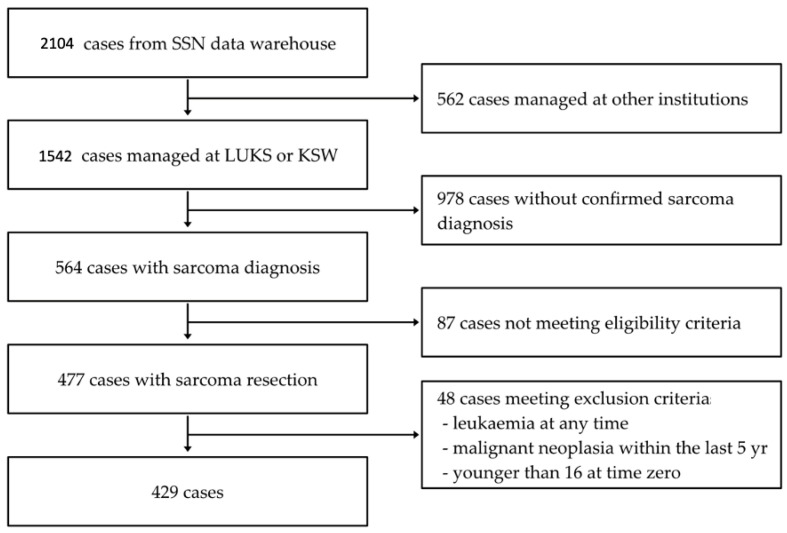
Flow chart.

As depicted in Figure 1, the SSN data warehouse hosts 2104 muskuloskeletal tumor cases registered by SSN members between 2018 and 2023. We included patients with a sarcoma diagnosis treated either as PEs or UEs and treated at Institution A or Institution B (n = 477) [9]. The inclusion criteria for this study encompassed all patients referred to our sarcoma center. Patients were included if they had a histologically confirmed diagnosis of sarcoma and had undergone surgical resection. The follow-up starts at the first surgical resection and ends at the occurrence of the end point (local recurrence, metastasis, or death), administrative censoring, or 5 years of follow-up. The median follow-up time was 1.9 years (1st quartile (Q1), 3rd quartile (Q3); 0.8 years, 3.7 years). Anatomical sites were categorized separately, including extremities, head and neck, trunk, and retroperitoneal regions, to ensure that site-specific outcomes were captured in our comparison of planned versus unplanned resections. Low-grade lesions, such as atypical lipomatous tumors (ALTs), were not excluded but were listed separately in the results to account for their different biological behavior.

Patients were excluded if they had a history of leukemia (n = 2), a malignant neoplasm within the last 5 years (n = 36), or were younger than 16 at time zero (n = 10). Additionally, the diagnosis of desmoid tumors was not included in this study population.

### 2.3. Study Design

The follow-up timeframe was defined from the initial surgical intervention (UE or PE) to predetermined outcomes (LR, metastasis, death) or to the date of the last patient contact. Logistic regression was used to ascertain the probability of UEs, with results represented by odds ratio (OR), 95% confidence interval (CI), and *p*-values. An OR above 1 indicated a higher likelihood of experiencing UEs, while an OR below 1 indicated a greater likelihood of PEs. A *p*-value below 0.05 was considered statistically significant. Variables included in the logistic regression analysis were sarcoma classification, anatomic region, size, grade, age, gender, Charlson comorbidity index (CCI), and diagnosis. Sensitivity analyses were conducted, encompassing additional statistical adjustments for additional variables such as various anatomic regions (head, neck, upper extremity, lower extremity, trunk, visceral, intraperitoneal, and retroperitoneal areas) and the five most common diagnoses (undifferentiated/unclassified sarcoma, atypical lipomatous tumor, myxoid liposarcoma, myxofibrosarcoma, and leiomyosarcoma) (see appendix). To account for the heterogeneity between soft tissue sarcomas and bone sarcomas, we stratified our analysis by sarcoma type. Separate statistical models were applied for each group, allowing for direct comparison while considering their distinct biological behaviors and treatment protocols. This approach provides a comprehensive yet focused analysis of the outcomes of planned versus unplanned resections for both sarcoma types. The management and characteristics of the UE group were characterized previously [9].

### 2.4. Statistical Analysis

The subset of UEs with LR was analyzed using both univariable and multivariable Cox regression models. The proportional hazards assumption for the Cox proportional hazards regression was examined using Schoenfeld residuals, and the linearity assumption for the covariates was assessed using Martingale residuals. In the multivariable Cox regression, Sarculator 5-year predictied survival was used to adjust for known prognostic factors due to the limited number of cases, necessitating a constraint on the number of variables [24]. Sarculator encompasses age, tumor size, FNCLCC grade, and histology, integrating multiple variables that would otherwise be analyzed separately. The 5-year Sarculator was chosen over the 10-year version based on the prevalence of five-year local recurrence-free survival rates reported in the existing literature. Myxoid liposarcoma was excluded from the Cox regression model due to its small sample size (only four patients in the UE group with one LR). We refrained from analyzing the subset of UEs with metastasis due to the limited sample size, rendering statistical analysis unfeasible (only nine patients with metastasis in the UE group).

### 2.5. Software and Ethical Considerations

Statistical analysis was conducted using the R statistical software (Version 4.3.2, Posit, PBC, Boston, MA, USA) [25]. This study was conducted in compliance with the Declaration of Helsinki and approved by the Ethics Committee of the Cantonal Ethics Committee Zurich, Switzerland (BASEC-Nr. 2019-01107/NCT04300257; 24 August 2021).

## 3. Results

We included 429 patients. Among them, 323 underwent PEs, while 106 experienced UEs. The baseline characteristics and outcomes of the study population, categorized into PE and UE treatment groups, are summarized in Table 1.

The tumor size showed substantial differences between the groups, with PEs associated with larger tumors (median 94 mm) compared to UEs (median 47 mm) (*p* < 0.001). Tumors in the upper extremity were more frequent in the UE group (19.8%) than in the PE group (6.2%) (*p* < 0.001). In contrast, lower extremity tumors were more often represented in the PE group (46.7%) than in the UE group (34%) (*p* = 0.02).

Regarding resection grading, a greater number of Grade 1 tumors were observed in the UE group (47.5%) compared to the PE group (30.2%) (*p* = 0.004). Conversely, the PE group had a higher proportion of Grade 3 tumors (50.5% vs. 37.4% for UE, *p* = 0.03).

R0 resection was more common in the PE group (78.8%) compared to the UE group (12.6%) (*p* < 0.001). According to the Sarculator 5-year prognostic tool, the 5-year survival rate was significantly higher in the UE group (median 87%) than in the PE group (median 68.5%) (*p* < 0.001).

Notably, metastasis rates at follow-up were higher in the PE group (31%) compared to the UE group (10.4%) (*p* < 0.001). Similarly, cancer-specific mortality was higher in the PE group (16.7%) compared to the UE group (6.6%) (*p* = 0.01). Despite the superior resection status in the PE group, these patients exhibited worse outcomes regarding metastasis at follow-up and cancer-specific mortality.

### 3.1. Variables to Predict the Likelihood of an UE

As shown in Table 2, several variables are associated with the likelihood of UEs. Sensitivity analyses (Appendix A Table A1, Table A2, Table A3, Table A4, Table A5, Table A6, Table A7, Table A8, Table A9, Table A10 and Table A11) confirmed that tumors situated in bone or deep soft tissue were less frequently associated with UEs, with an OR of 0.13 (95% CI: 0.04, 0.44; *p* = 0.001) for bone tumors and an OR of 0.33 (95% CI: 0.16; 0.66; *p* = 0.002) for deep soft tissue tumors, indicating a significant reduction in the association of these tumor types with UEs compared to superficial tumors. Tumor localization also played a critical role, with tumors in the upper extremity more likely to undergo UEs. Specifically, axial tumors had an OR of 0.32 (CI: 0.12, 0.84; *p* = 0.02) and lower extremity tumors had an OR of 0.33 (95% CI: 0.13, 0.83, *p* = 0.02), suggesting a strong association with UEs.

Tumor size significantly influenced the probability of UEs. Tumors measuring between 5 cm and 10 cm had an OR of 0.52 (95% CI: 0.27, 1.00; *p* = 0.05), while tumors larger than 10 cm had an OR of 0.16 (95 CI: 0.06, 0.41; *p* < 0.001), indicating that larger tumors are more often associated with PEs. Tumor grade similarly affected the likelihood of UEs, with Grade 2 tumors showing an OR of 0.13 (95% CI: 0.04, 0.36; *p* < 0.001) and Grade 3 tumors having an OR of 0.21 (95% CI: 0.09, 0.46; *p* < 0.001), suggesting that higher-grade tumors are more likely to be planned resections.

No age-related influence was observed (OR = 0.99; 95% CI: 0.96, 1.03; *p* = 0.67). However, gender was associated with a higher likelihood of UEs, with men having higher odds of UEs (OR = 2.11; 95% CI: 1.19, 3.84; *p* = 0.01). The Charlson comorbidity index (CCI) was not specifically linked to either UEs or PEs (OR = 1.06; 95% CI: 0.75, 1.48; *p* = 0.75).

Among the five most common diagnoses, myxoid liposarcoma was associated with PEs (OR = 0.20; 95% CI: 0.04, 0.81; *p* = 0.04), whereas leiomyosarcoma demonstrated a significant association with UEs (OR = 2.96; 95% CI: 1.14, 7.76; *p* = 0.03).

The sensitivity analysis (see Appendix) showed no substantial impact on the results apart from the diagnosis of myxofibrosarcoma, which varies in its result depending on the tumor’s location. Myxofibrosacoma reached statistical significance when used with the location of head and neck, lower extremity, trunk, retroperitoneal location, and visceral and intraperitoneal location. It does not display significant results when combined with the anatomical location of the upper extremity.

### 3.2. Independent Predictors of Local Recurrence-Free Survival in the UE Subset

The results, presented in Table 3, indicate that tumor sizes ranging between 5 cm and 10 cm (HR 2.89, 95% CI: 1.06, 7.87; *p* = 0.04), Grade 3 tumors (HR 2.97, 95% CI: 1.11, 7.94; *p* = 0.03), age (HR 1.04, 95% CI: 1.01, 1.07; *p* = 0.003), and CCI (HR 1.47, 95% CI: 1.13, 1.92; *p* = 0.004) were significantly associated with LR-free survival in the UE subset. Sensitivity analyses (Appendix A, Table A1, Table A2, Table A3, Table A4, Table A5, Table A6, Table A7, Table A8, Table A9, Table A10 and Table A11) consistently supported these findings, highlighting the impact of these parameters across different tumor locations. As individual predictors, sarcoma classification, anatomical region, gender, and diagnosis do not influence local recurrence-free survival.

Table 4 shows that the Sarculator 5-year survival prediction remains a significant predictor of local recurrence-free survival in unplanned excisions (UEs). Used in this analysis were the variables sarcoma classification, anatomic region, size, grade, age, gender, cci, diagnosis, and Sarculator. Sarculator remains a significant variable, whereas the single variables showed no significance. This underscores Sarculator’s utility as a reliable tool for predicting recurrence-free survival, even when individual variables show no significance.

## 4. Discussion

Our study demonstrates that PEs are associated with larger, higher-grade tumors and better immediate surgical outcomes (R0 status) but also exhibit higher rates of metastasis and cancer-specific mortality compared to UEs. PEs typically involve tumors at a more advanced stage and with more aggressive characteristics, which contribute to poorer long-term outcomes. In contrast, UEs often involve smaller, less aggressive tumors detected earlier, leading to lower metastatic potential despite higher LR rates. These findings underscore the significant differences in tumor and patient characteristics, surgical outcomes, and prognosis between PEs and UEs.

In our previous paper, we reported that UEs were associated with higher LR rates but found no significant differences in MFS and OS between UEs and PEs [9]. This suggested that while UEs compromised local control due to suboptimal initial surgical margins, they did not necessarily impact the long-term risk of metastasis or OS. Our current study expands on these findings by demonstrating that PEs are associated with higher metastasis rates and cancer-specific mortality compared to UEs. This discrepancy can be attributed to the larger and more advanced nature of tumors typically encountered in PEs, which inherently increases the risk of distant spread and mortality despite achieving better local control with R0 resection margins. The existing literature on the relationship between UEs and metastatic risk is diverse and sometimes contradictory [26]. Several studies corroborate our initial findings, indicating that UEs primarily increase LR rates without significantly affecting MFS or OS [16,19,27,28]. Conversely, other studies suggest a potential link between UEs and increased metastatic risk, positing that inadequate initial surgery may facilitate metastatic progression [17,18,29]. Our current findings contribute to this debate by providing evidence that the advanced stage and larger size of tumors in the PE group are critical factors driving higher metastasis rates and cancer-specific mortality. These observations underscore the importance of early detection and timely referral to specialized sarcoma hubs to prevent the progression of sarcomas to advanced stages that necessitate PEs, thereby reducing the associated metastatic risk. In summary, while our predecessor paper and some studies suggest that UEs mainly compromise local control, our current findings emphasize that the characteristics of tumors requiring PEs, such as larger size and higher grade, are pivotal in determining metastatic potential and cancer-specific mortality, highlighting the need for strategies aimed at early detection and specialized intervention to improve patient outcomes [9].

The findings from our study have several important implications for clinical practice. The significant association between PEs and higher rates of metastasis and cancer-specific mortality underscores the necessity of early detection and timely intervention. However, early detection alone does not always guarantee a reduction in unplanned surgeries (whoops). Reducing the likelihood of UEs depends not only on diagnostic imaging but also on high clinical suspicion, standardized diagnostic protocols, and early referral to specialized sarcoma hubs. A multidisciplinary approach, involving experienced teams, ensures more accurate diagnoses and improves surgical planning, ultimately minimizing the incidence of unplanned surgeries [11]. To reduce the incidence of UEs, clinicians should maintain a high index of suspicion for sarcomas, especially in patients presenting with suspicious soft tissue masses. Implementing standardized diagnostic protocols, including advanced imaging techniques and biopsy procedures, can aid in accurate diagnosis before any surgical intervention. Educational initiatives aimed at primary care physicians and general surgeons are essential to enhance early detection and appropriate referral practices. By increasing awareness and knowledge about sarcomas, these healthcare providers can better identify potential cases and refer patients to specialized sarcoma hubs sooner. Integrating AI-prediction tools into clinical workflows can help identify high-risk cases and facilitate personalized treatment planning. Moreover, there is a critical need for advanced diagnostic tools that can accurately differentiate between sarcomas and benign tumors. Developing and integrating such risk assessment tools would enable clinicians to better determine the likelihood of malignancy in suspicious cases, leading to more timely and appropriate interventions. These tools can analyze patient data to flag potential sarcoma cases for further investigation, ensuring that appropriate therapeutic measures are taken promptly. AI models can improve diagnostic accuracy, predict outcomes more effectively, and tailor interventions to individual patient profiles. Multidisciplinary tumor boards should review sarcoma cases to formulate comprehensive, individualized treatment plans that consider all relevant factors, including tumor size, grade, and anatomical location. This collaborative approach ensures that each patient receives a tailored treatment strategy designed to optimize outcomes. By focusing on early detection and appropriate surgical planning, we can reduce the incidence of UEs and improve long-term outcomes for patients with sarcomas. Early identification of sarcomas allows for PEs, which are associated with better immediate surgical outcomes and potentially lower rates of metastasis and cancer-specific mortality. Adopting these practices will significantly enhance patient care and survival rates.

Future research should focus on creating and validating these tools to predict high-risk cases accurately. By integrating individual tumor and patient characteristics, these models can enhance early detection, optimize treatment planning, and personalize therapeutic interventions. Additionally, studies exploring the implementation of standardized diagnostic protocols and their impact on reducing UEs could provide valuable insights. Investigating the long-term outcomes of patients with sarcomas managed with these advanced predictive tools and standardized protocols will help assess their effectiveness in real-world/time clinical settings. Research should also aim to better understand the genetic and molecular profiles of sarcomas, which can contribute to more targeted and effective treatment strategies. Longitudinal studies tracking the progression of sarcomas from diagnosis through treatment and follow-up could further elucidate the factors influencing metastasis and survival.

Our study has several limitations. Although the data were collected as real-world/time prospective data with retrospective review and control, potential biases such as selection bias and information bias may still be present. The accuracy of medical records can vary, potentially affecting the reliability of our findings. The study population, limited to patients treated at specialized sarcoma hubs, may not be representative of the broader population, affecting the generalizability of our findings to other settings with different patient demographics or healthcare resources. Variability in surgical techniques and postoperative care among different surgeons and institutions can influence outcomes, and although we attempted to control for these factors, residual confounding may still exist. Additionally, this study did not account for all possible variables influencing outcomes, such as genetic factors, detailed patient comorbidities beyond the Charlson Comorbidity Index, and specific treatment regimens, which may affect sarcoma prognosis. The follow-up period may not be sufficient to capture all long-term outcomes, particularly late metastasis and OS, necessitating longer follow-up studies to confirm the durability of observed outcomes and provide a comprehensive understanding of the long-term implications of PEs versus UEs. While our study highlights key factors influencing sarcoma outcomes and suggests the potential benefits of AI prediction tools, further research is needed to develop and validate these tools in diverse clinical settings. Prospective studies with larger, more diverse populations and standardized protocols are necessary to confirm our findings and enhance the applicability of AI models in clinical practice. Despite these limitations, our study provides valuable insights into the differences between PEs and UEs and underscores the importance of early detection and appropriate referral to specialized sarcoma hubs. Future research should aim to address these limitations and further explore the potential of advanced predictive tools in improving sarcoma management.

## 5. Conclusions

Our study demonstrates that PEs achieve superior immediate surgical outcomes (R0 resection) but are linked to higher metastasis and cancer-specific mortality due to the advanced stage of tumors. Conversely, UEs, while associated with higher LR rates, do not significantly affect MFS or OS. These findings, building on our predecessor study, highlight the critical role of tumor characteristics in long-term outcomes. Early detection, comprehensive diagnostics, and timely referrals to specialized sarcoma hubs are essential to avoid UEs and reduce metastatic risk. This study addresses the ongoing literature debate on UEs’ impact, emphasizing that advanced tumor features primarily drive poor prognosis. Variations in study populations, treatment modalities, and parameter distributions can explain contradictory findings in the literature. Future research should focus on developing predictive tools using individual tumor and patient characteristics to improve sarcoma management. This includes the creation and validation of AI-based models to accurately predict cases with a high risk of sarcoma diagnosis, which can facilitate early detection and personalized surgical planning, ultimately improving patient outcomes.

## Figures and Tables

**Table 1:** Characteristics of the study population categorized by treatment groups.

	All	PEs	UEs	*p*-Value
**n**	429	323	106	-
**Gender**				
Male, n (%)	229 (53.4)	167 (51.7)	62 (58.5)	0.26
Female, n (%)	200 (46.6)	156 (48.3)	44 (41.5)	0.26
**Age in years**, median (Q1, Q3)	63 (51, 74)	63 (53, 74)	63.5 (47, 75)	0.45
**Sarcoma classification**				
SST, n (%)	69 (16.1)	31 (9.6)	38 (35.8)	<0.001
DST, n (%)	317 (73.9)	256 (79.3)	61 (57.5)	<0.001
Bone, n (%)	43 (10.0)	36 (11.1)	7 (6.6)	0.20
**Diagnosis** *				
Myxoid liposarcoma, n (%)	28 (6.5)	24 (7.4)	4 (3.8)	0.26
Dedifferentiated liposarcoma, n (%)	40 (9.3)	36 (11.1)	4 (3.8)	0.02
Leiomyosarcoma, n (%)	47 (11.0)	31 (9.6)	16 (15.1)	0.15
Atypical lipomatous tumor	63 (14.7)	51 (15.8)	12 (11.3)	0.34
Undifferentiated/unclassified sarcoma	64 (14.9)	52 (15.8)	12 (11.3)	0.27
**Tumor size** (largest diameter) in mm, median (Q1, Q3)	80 (50, 129)	94 (60, 143)	46.5 (27, 79)	<0.001
**Anatomic region**				
Upper extremity, n (%)	41 (9.6)	20 (6.2)	21 (19.8)	<0.001
Axial, n (%)	201 (46.9)	152 (47.1)	49 (46.2)	0.91
Lower extremity, n (%)	187 (43.6)	151 (46.7)	36 (34.0)	0.02
**Resection grading** (time zero)				
G1, n (%)	138 (34.5)	91 (30.2)	47 (47.5)	0.004
G2, n (%)	73 (18.3)	58 (19.3)	15 (15.2)	0.46
G3, n (%)	189 (47.3)	152 (50.5)	37 (37.4)	0.03
**Resection status** (time zero) ^†^				
R0, n (%)	253 (63.1)	241 (78.8)	12 (12.6)	<0.001
R1/R2, n (%)	148 (36.9)	65 (21.2)	83 (87.4)	<0.001
**CCI**, median (Q1, Q3)	2 (0, 3)	2 (1, 3)	1 (0, 3)	0.15
**Sarculator 5 years** in %**,** median (Q1, Q3)	73 (54, 89)	68.5 (50, 83)	87 (73, 93)	<0.001
**Local recurrence**, n (%)	82 (19.1)	56 (17.3)	26 (24.5)	0.27
**Metastasis at diagnosis**, n (%)	23 (5.4)	20 (6.2)	3 (2.8)	0.22
**Metastasis at follow-up**, n (%)	111 (25.9)	100 (31.0)	11 (10.4)	<0.001
**Death**				
Overall, n (%)	71 (16.6)	61 (18.9)	10 (9.4)	0.25
Cancer specific, n (%)	61 (14.2)	54 (16.7)	7 (6.6)	0.01

The percentages correspond to the number of cases (n-values) listed in the first row of their respective columns. * The listing comprises only five common diagnoses within our study cohort. ^†^ Histological examination determines the R0 status. In contrast, a negative postoperative MRI finding characterizes R1 status, unlike R2. To distinguish between R1 and R2 statuses, the surgical report was also assessed.

**Table 2:** Logistic regression analysis of predictor variables for UEs.

Characteristics	OR	95% CI	*p*-Value
**Sarcoma classification**			
SST	—	—	
Bone	0.13	0.04, 0.44	0.001
DST	0.33	0.16, 0.66	0.002
**Anatomic region**			
Upper extremity	—	—	
Axial	0.32	0.12, 0.84	0.02
Lower extremity	0.33	0.13, 0.83	0.02
**Size**			
<5 cm	—	—	
5–10 cm	0.52	0.27, 1.00	0.05
>10 cm	0.16	0.06, 0.41	<0.001
**Grade**			
1	—	—	
2	0.13	0.04, 0.36	<0.001
3	0.21	0.09, 0.46	<0.001
**Age**	0.99	0.96, 1.03	0.67
**Gender**			
Female	—	—	
Male	2.11	1.19, 3.84	0.01
**CCI**	1.06	0.75, 1.48	0.75
**Diagnosis**			
Rest	—	—	
Undifferentiated/unclassified sarcoma	1.12	0.42, 2.93	0.82
Atypical lipomatous tumor	0.48	0.15, 1.48	0.21
Myxoid liposarcoma	0.20	0.04, 0.81	0.04
Myxofibrosarcoma	1.99	0.63, 6.27	0.24
Leiomyosarcoma (excluding skin)	2.96	1.14, 7.76	0.03

**Table 3:** Univariable Cox regression for local recurrence-free survival.

Characteristics	HR	95% CI	*p*-Value
**Sarcoma classification**			
SST	—	—	
Bone	1.74	0.33, 9.14	0.51
DST	1.89	0.69, 5.17	0.22
**Anatomic region**			
Upper extremity	—	—	
Axial	1.58	0.56, 4.42	0.39
Lower extremity	0.61	0.18, 2.11	0.43
**Size**			
<5 cm	—	—	
5–10 cm	2.89	1.06, 7.87	0.04
>10 cm	2.86	0.78, 10.56	0.11
**Grade**			
1	—	—	
2	1.99	0.56, 7.12	0.29
3	2.97	1.11, 7.94	0.03
**Age**	1.04	1.01, 1.07	0.003
**Gender**			
Female	—	—	
Male	0.94	0.42, 2.11	0.89
**CCI**	1.47	1.13, 1.92	0.004
**Diagnosis**			
Rest	—	—	
Undifferentiated/unclassified sarcoma	1.75	0.47, 6.53	0.41
Atypical lipomatous tumor	1.12	0.37, 3.34	0.84
Myxofibrosarcoma	1.56	0.49, 4.94	0.45
Leiomyosarcoma (excluding skin)	0.67	0.15, 3.03	0.60

**Table 4:** Multivariable Cox regression, Sarculator 5-year survival as reference.

Characteristics	HR	95% CI	*p*-Value	Sarculator 5y, HR	Sarculator 5y, 95% CI	Sarculator 5y, *p*-Value
**Sarcoma classification**				0.02	0.001, 0.24	0.003
SST	—	—				
Bone	1.50	0.26, 8.58	0.65			
DST	1.12	0.33, 3.82	0.86			
**Anatomic region**				0.02	0.002, 0.25	0.002
Upper extremity	—	—				
Axial	1.07	0.30, 3.80	0.92			
Lower extremity	0.72	0.17, 2.97	0.65			
**Size**				0.08	0.01, 1.30	0.08
<5 cm	—	—				
5–10 cm	2.50	0.69, 9.07	0.16			
>10 cm	5.60	0.62, 50.88	0.13			
**Grade**				0.03	0.002, 0.54	0.02
1	—	—				
2	1.09	0.18, 6.75	0.92			
3	1.51	0.32, 7.05	0.60			
**Age**	1.02	0.99, 1.05	0.21	0.02	0.002, 0.29	0.003
**Gender**				0.009	0.0007, 0.11	0.0003
Female	—	—				
Male	0.48	0.16, 1.44	0.19			
**CCI**	1.25	0.87, 1.78	0.23	0.02	0.00, 0.29	0.003
**Diagnosis**				0.02	0.00, 0.15	0.0003
Rest	—	—				
Undifferentiated/unclassified sarcoma	1.37	0.34, 5.45	0.66			
Atypical lipomatous tumor						
Myxofibrosarcoma	1.02	0.29, 3.57	0.97			
Leiomyosarcoma (excluding skin)	0.22	0.03, 1.82	0.16			

## Data Availability

The data presented in this study are available on request from the corresponding author.

## Appendix A

**Table A1:** Sensitivity analysis, head and neck—logistic regression.

Characteristics	OR	95% CI	*p*-Value
**Sarcoma classification**			
SST	—	—	
Bone	0.18	0.05, 0.56	0.004
DST	0.36	0.18, 0.72	0.004
**Anatomic region**			
Head and neck	—	—	
Rest	0.86	0.22, 3.40	0.82
**Size**			
<5 cm	—	—	
5–10 cm	0.52	0.27, 1.00	0.05
>10 cm	0.15	0.06, 0.37	<0.001
**Grade**			
1	—	—	
2	0.14	0.05, 0.38	<0.001
3	0.21	0.09, 0.47	<0.001
**Age**	0.99	0.96, 1.02	0.56
**Gender**			
Female	—	—	
Male	1.95	1.11, 3.49	0.02
**CCI**	1.05	0.75, 1.46	0.76
**Diagnosis**			
Rest	—	—	
Undifferentiated/unclassified sarcoma	1.38	0.54, 3.45	0.50
Atypical lipomatous tumor	0.51	0.16, 1.52	0.23
Myxoid liposarcoma	0.20	0.04, 0.77	0.03
Myxofibrosarcoma	3.41	1.22, 9.69	0.02
Leiomyosarcoma (excluding skin)	3.01	1.17, 7.81	0.02

**Table A2:** Sensitivity analysis, upper extremity—logistic regression.

Characteristics	OR	95% CI	*p*-Value
**Sarcoma classification**			
SST	—	—	
Bone	0.13	0.04, 0.43	0.001
DST	0.33	0.16, 0.66	0.002
**Anatomic region**			
Rest	—	—	
Upper extremity	3.08	1.27, 7.54	0.01
**Size**			
<5 cm	—	—	
5–10 cm	0.51	0.27, 0.99	0.05
>10 cm	0.16	0.06, 0.41	<0.001
**Grade**			
1	—	—	
2	0.13	0.04, 0.36	<0.001
3	0.21	0.09, 0.47	<0.001
**Age**	0.99	0.96, 1.03	0.67
**Gender**			
Female	—	—	
Male	2.11	1.19, 3.81	0.01
**CCI**	1.06	0.75, 1.48	0.75
**Diagnosis**			
Rest	—	—	
Undifferentiated/unclassified sarcoma	1.13	0.44, 2.87	0.80
Atypical lipomatous tumor	0.48	0.16, 1.47	0.20
Myxoid liposarcoma	0.20	0.04, 0.77	0.03
Myxofibrosarcoma	2.02	0.67, 6.11	0.21
Leiomyosarcoma (excluding skin)	2.96	1.14, 7.75	0.03

**Table A3:** Sensitivity analysis, lower extremity—logistic regression.

Characteristics	OR	95% CI	*p*-Value
**Sarcoma classification**			
SST	—	—	
Bone	0.19	0.06, 0.61	0.006
DST	0.36	0.18, 0.71	0.004
**Anatomic region**			
Lower extremity	—	—	
Rest	1.31	0.72, 2.41	0.38
**Size**			
<5 cm	—	—	
5–10 cm	0.50	0.26, 0.96	0.04
>10 cm	0.15	0.06, 0.37	<0.001
**Grade**			
1	—	—	
2	0.15	0.05, 0.40	<0.001
3	0.21	0.09, 0.47	<0.001
**Age**	0.99	0.96, 1.02	0.53
**Gender**			
Female	—	—	
Male	1.93	1.10, 3.46	0.02
**CCI**	1.05	0.75, 1.46	0.76
**Diagnosis**			
Rest	—	—	
Undifferentiated/unclassified sarcoma	1.47	0.57, 3.74	0.42
Atypical lipomatous tumor	0.55	0.18, 1.69	0.30
Myxoid liposarcoma	0.23	0.04, 0.96	0.06
Myxofibrosarcoma	3.53	1.27, 10.0	0.02
Leiomyosarcoma (excluding skin)	2.97	1.16, 7.66	0.02

**Table A4:** Sensitivity analysis, trunk—logistic regression.

Characteristics	OR	95% CI	*p*-Value
**Sarcoma classification**			
SST	—	—	
Bone	0.17	0.05, 0.55	0.004
DST	0.34	0.17, 0.70	0.003
**Anatomic region**			
Rest	—	—	
Trunk	0.90	0.42, 1.87	0.79
**Size**			
<5 cm	—	—	
5–10 cm	0.52	0.27, 1.01	0.05
>10 cm	0.15	0.06, 0.37	<0.001
**Grade**			
1	—	—	
2	0.14	0.05, 0.38	<0.001
3	0.21	0.09, 0.46	<0.001
**Age**	0.99	0.96, 1.02	0.60
**Gender**			
Female	—	—	
Male	1.97	1.13, 3.52	0.02
**CCI**	1.05	0.74, 1.45	0.79
**Diagnosis**			
Rest	—	—	
Undifferentiated/unclassified sarcoma	1.35	0.53, 3.36	0.52
Atypical lipomatous tumor	0.50	0.16, 1.50	0.22
Myxoid liposarcoma	0.19	0.04, 0.76	0.03
Myxofibrosarcoma	3.29	1.18, 9.33	0.02
Leiomyosarcoma (excluding skin)	2.93	1.14, 7.66	0.03

**Table A5:** Sensitivity analysis, retroperitoneal—logistic regression.

Characteristics	OR	95% CI	*p*-Value
**Sarcoma classification**			
SST	—	—	
Bone	0.17	0.05, 0.54	0.003
DST	0.38	0.19, 0.75	0.006
**Anatomic region**			
Rest	—	—	
Retroperitoneal	0.44	0.12, 1.30	0.17
**Size**			
<5 cm	—	—	
5–10 cm	0.53	0.28, 1.02	0.06
>10 cm	0.16	0.06, 0.39	<0.001
**Grade**			
1	—	—	
2	0.15	0.05, 0.41	<0.001
3	0.22	0.10, 0.49	<0.001
**Age**	0.99	0.96, 1.02	0.58
**Gender**			
Female	—	—	
Male	1.99	1.13, 3.56	0.02
**CCI**	1.07	0.76, 1.48	0.71
**Diagnosis**			
Rest	—	—	
Undifferentiated/unclassified sarcoma	1.20	0.47, 3.02	0.70
Atypical lipomatous tumor	0.48	0.15, 1.45	0.20
Myxoid liposarcoma	0.19	0.04, 0.72	0.03
Myxofibrosarcoma	3.02	1.09, 8.56	0.04
Leiomyosarcoma (excluding skin)	3.15	1.21, 8.30	0.02

**Table A6:** Sensitivity analysis, visceral and intraperitoneal—logistic regression.

Characteristics	OR	95% CI	*p*-Value
**Sarcoma classification**			
SST	—	—	
Bone	0.18	0.05, 0.55	0.004
DST	0.35	0.17, 0.70	0.003
**Anatomic region**			
Rest	—	—	
Visceral and intraperitoneal	1.05	0.38, 2.65	0.92
**Size**			
<5 cm	—	—	
5–10 cm	0.52	0.27, 0.99	0.05
>10 cm	0.15	0.06, 0.37	<0.001
**Grade**			
1	—	—	
2	0.14	0.05, 0.38	<0.001
3	0.21	0.09, 0.47	<0.001
**Age**	0.99	0.96, 1.02	0.58
**Gender**			
Female	—	—	
Male	1.96	1.12, 3.51	0.02
**CCI**	1.05	0.75, 1.46	0.77
**Diagnosis**			
Rest	—	—	
Undifferentiated/unclassified sarcoma	1.36	0.54, 3.40	0.51
Atypical lipomatous tumor	0.50	0.16, 1.51	0.22
Myxoid liposarcoma	0.20	0.04, 0.77	0.03
Myxofibrosarcoma	3.37	1.21, 9.60	0.02
Leiomyosarcoma (excluding skin)	2.95	1.13, 7.87	0.03

**Table A7:** Sensitivity analysis, undifferentiated/unclassified sarcoma—logistic regression.

Characteristics	OR	95% CI	*p*-Value
**Sarcoma classification**			
SST	—	—	
Bone	0.15	0.05, 0.45	<0.001
DST	0.34	0.17, 0.66	0.001
**Anatomic region**			
Upper extremity	—	—	
Axial	0.30	0.13, 0.70	0.006
Lower extremity	0.26	0.11, 0.61	0.002
**Size**			
<5 cm	—	—	
5–10 cm	0.40	0.21, 0.73	0.003
>10 cm	0.11	0.05, 0.26	<0.001
**Grade**			
1	—	—	
2	0.30	0.13, 0.67	0.004
3	0.37	0.19, 0.73	0.004
**Age**	1.00	0.97, 1.03	0.90
**Gender**			
Female	—	—	
Male	1.84	1.06, 3.23	0.03
**CCI**	1.01	0.72, 1.41	0.94
**Diagnosis**			
Undifferentiated/unclassified sarcoma	—	—	
Rest	1.18	0.51, 2.84	0.71

**Table A8:** Sensitivity analysis, atypical lipomatous tumor—logistic regression.

Characteristics	OR	95% CI	*p*-Value
**Sarcoma classification**			
SST	—	—	
Bone	0.14	0.04, 0.43	<0.001
DST	0.35	0.18, 0.67	0.002
**Anatomic region**			
Upper extremity	—	—	
Axial	0.30	0.13, 0.71	0.006
Lower extremity	0.26	0.11, 0.62	0.002
**Size**			
<5 cm	—	—	
5–10 cm	0.41	0.22, 0.76	0.005
>10 cm	0.13	0.05, 0.31	<0.001
**Grade**			
1	—	—	
2	0.26	0.11, 0.61	0.003
3	0.31	0.15, 0.63	0.001
**Age**	1.00	0.97, 1.04	0.82
**Gender**			
Female	—	—	
Male	1.87	1.08, 3.31	0.03
**CCI**	1.01	0.72, 1.40	0.97
**Diagnosis**			
Atypical lipomatous tumor	—	—	
Rest	1.58	0.57, 4.54	0.38

**Table A9:** Sensitivity analysis, myxoid liposarcoma—logistic regression.

Characteristics	OR	95% CI	*p*-Value
**Sarcoma classification**			
SST	—	—	
Bone	0.14	0.04, 0.40	<0.001
DST	0.34	0.17, 0.66	0.002
**Anatomic region**			
Upper extremity	—	—	
Axial	0.29	0.12, 0.68	0.004
Lower extremity	0.28	0.12, 0.68	0.005
**Size**			
<5 cm	—	—	
5–10 cm	0.45	0.24, 0.84	0.01
>10 cm	0.11	0.05, 0.26	<0.001
**Grade**			
1	—	—	
2	0.27	0.11, 0.60	0.002
3	0.31	0.16, 0.59	<0.001
**Age**	1.00	0.97, 1.03	0.83
**Gender**			
Female	—	—	
Male	1.92	1.10, 3.39	0.02
**CCI**	1.03	0.73, 1.43	0.85
**Diagnosis**			
Myxoid liposarcoma	—	—	
Rest	4.01	1.08, 20.0	0.06

**Table A10:** Sensitivity analysis, myxofibrosarcoma—logistic regression.

Characteristics	OR	95% CI	*p*-Value
**Sarcoma classification**			
SST	—	—	
Bone	0.17	0.05, 0.51	0.002
DST	0.35	0.18, 0.69	0.002
**Anatomic region**			
Upper extremity	—	—	
Axial	0.37	0.15, 0.93	0.03
Lower extremity	0.29	0.12, 0.73	0.01
**Size**			
<5 cm	—	—	
5–10 cm	0.39	0.21, 0.73	0.003
>10 cm	0.12	0.05, 0.27	<0.001
**Grade**			
1	—	—	
2	0.30	0.13, 0.67	0.004
3	0.35	0.18, 0.65	0.001
**Age**	1.00	0.97, 1.03	0.98
**Gender**			
Female	—	—	
Male	1.80	1.04, 3.17	0.04
**CCI**	1.01	0.72, 1.40	0.94
**Diagnosis**			
Myxofibrosarcoma	—	—	
Rest	0.56	0.20, 1.54	0.26

**Table A11:** Sensitivity analysis, leiomyosarcoma—logistic regression.

Characteristics	OR	95% CI	*p*-Value
**Sarcoma classification**			
SST	—	—	
Bone	0.16	0.05, 0.46	0.001
DST	0.32	0.16, 0.62	<0.001
**Anatomic region**			
Upper extremity	—	—	
Axial	0.27	0.11, 0.64	0.003
Lower extremity	0.25	0.10, 0.59	0.002
**Size**			
<5 cm	—	—	
5–10 cm	0.42	0.22, 0.78	0.01
>10 cm	0.12	0.05, 0.28	<0.001
**Grade**			
1	—	—	
2	0.20	0.07, 0.49	<0.001
3	0.34	0.18, 0.64	<0.001
**Age**	1.00	0.97, 1.03	0.98
**Gender**			
Female	—	—	
Male	1.96	1.12, 3.48	0.02
**CCI**	1.03	0.73, 1.44	0.85
**Diagnosis**			
Leiomyosarcoma (excluding skin)	—	—	
Rest	0.37	0.15, 0.90	0.03
